# Microglia knockdown reduces inflammation and preserves cognition in diabetic animals after experimental stroke

**DOI:** 10.1186/s12974-020-01815-3

**Published:** 2020-04-28

**Authors:** Ladonya Jackson, Selin Dumanli, Maribeth H. Johnson, Susan C. Fagan, Adviye Ergul

**Affiliations:** 1 Program in Clinical and Experimental Therapeutics, University of Georgia College of Pharmacy, Augusta, GA USA; 2 Charlie Norwood VA Medical Center, Augusta, GA USA; 3 Department of Pathology and Laboratory Medicine, Medical University of South Carolina, 171 Ashley Ave. MSC, Charleston, SC USA; 4 Department of Pathology and Laboratory Medicine, Ralph H. Johnson Veterans Affairs Medical Center, Charleston, SC USA; 5 Department Neuroscience and Regenerative Medicine, Medical College of Georgia, Augusta University, Augusta, GA USA; 6 Department of Physiology, Medical College of Georgia, Augusta University, Augusta, GA USA

**Keywords:** Microglia, shRNA, Colony stimulating factor 1 receptor CSF1R, Diabetes, Post-stroke cognitive impairment

## Abstract

**Introduction:**

Unfortunately, over 40% of stroke victims have pre-existing diabetes which not only increases their risk of stroke up to 2–6 fold, but also worsens both functional recovery and the severity of cognitive impairment. Our lab has recently linked the chronic inflammation in diabetes to poor functional outcomes and exacerbated cognitive impairment, also known as post-stroke cognitive impairment (PSCI). Although we have shown that the development of PSCI in diabetes is associated with the upregulation and the activation of pro-inflammatory microglia, we have not established direct causation between the two. To this end, we evaluated the role of microglia in the development of PSCI.

**Methods:**

At 13 weeks of age, diabetic animals received bilateral intracerebroventricular (ICV) injections of short hairpin RNA (shRNA) lentiviral particles targeting the colony stimulating factor 1 receptor (CSF1R). After 14 days, animals were subjected to 60 min middle cerebral artery occlusion (MCAO) or sham surgery. Adhesive removal task (ART), novel object recognition (NOR), and 2-trial Y-maze were utilized to evaluate sensorimotor and cognitive function. Tissue from freshly harvested brains was analyzed by flow cytometry and immunohistochemistry.

**Results:**

CSF1R silencing resulted in a 94% knockdown of residential microglia to relieve inflammation and improve myelination of white matter in the brain. This prevented cognitive decline in diabetic animals.

**Conclusion:**

Microglial activation after stroke in diabetes may be causally related to the development of delayed neurodegeneration and PSCI.

## Introduction

Post-stroke cognitive impairment (PSCI), the rate of which doubled between 1990 and 2000, likely due to decreased stroke mortality [1], is an important component of vascular contributions to cognitive impairment and dementia (VCID) spectrum disorders [2]. PSCI, along with sensorimotor deficits that develop after the ischemic brain injury, makes stroke a major cause of adult disability with more than 60% of its victims struggling to perform daily activities and losing their independence [3]. As the rate of both stroke and PSCI increases with our aging population, there is an urgent need for better understanding of the mechanisms that hamper or promote recovery after stroke in order to identify therapeutic targets and strategies [4].

It is recognized that the balance of pro- and anti-inflammatory mediators determines the detrimental or beneficial effects of neuroinflammation on brain injury and repair after stroke [5, 6]. This balance is altered in favor of pro-inflammatory cells and cytokines in comorbid diseases like diabetes [7, 8]. Microglia, as the resident immune cells of the brain, exist in three functional phenotypes known as surveillance (M0), pro-inflammatory, and anti-inflammatory states and can dynamically change from one form to another [9, 10]. In experimental stroke, microglia are activated to an anti-inflammatory state in the acute phase of stroke but they are rapidly polarized to an pro-inflammatory state by the ischemic neurons [6]. However, this early anti-inflammatory state is absent when similar experiments are conducted in diabetic animals and this is associated with impaired white matter repair and long-term functional deficits [11]. We also showed that diabetes, as a chronic inflammatory disease, increases the number of activated ameboid microglia and worsens cognitive outcomes of stroke [12, 13]. These studies suggest that the inflammatory status of microglia can be modulated by the microenvironment [14]. Given that 70% of stroke victims have pre-existing comorbidities such as diabetes and hypertension at the time of stroke, and diabetes worsens functional recovery and the severity of PSCI to an even greater extent, better understanding of the “primed” inflammatory microglia in the brain may allow us to modulate these cells to a beneficial phenotype to prevent progression of PSCI [8, 15].

Our lab recently linked the chronically elevated pro-inflammatory/anti-inflammatory ratio observed in diabetic animals to the development of PSCI [16]. The predominance of pro-inflammatory microglia was accompanied by increased astrocyte activation and demyelination of the hippocampus, an important region for learning and memory. When this ratio was modulated with therapeutic intervention using an angiotensin II type 2 receptor (AT2R) agonist, the number of astrocytes was reduced, myelination of the hippocampus was preserved, and the development of PSCI was prevented [16]. Although we have shown that the development of diabetic PSCI is associated with the upregulation and the activation of pro-inflammatory microglia, we have not established a causal link between the two. Also, the exact role of microglia activation in functional recovery has not been fully elucidated. Anti-inflammatory M2 microglia aid in the remodeling of synapses and secrete growth factors such as brain-derived neurotrophic factor (BDNF) [17]. The depletion of microglia-derived BDNF is associated with altered synaptic protein levels and impaired spine formation during motor learning tasks, which has been shown to result in the absence of training-induced improvement in motor performance [18]. As discussed above, the acute rise in anti-inflammatory microglia to aid recovery after stroke in control animals [6] is absent in diabetic animals [11]. These findings led us to question whether the depletion of microglia would alleviate (1) chronic neuroinflammation and (2) the development of PSCI and functional deficits in diabetic animals, since the normal repair processes facilitated by anti-inflammatory microglia are already blunted.

To address these questions, we used a molecular approach to eliminate microglia. Colony-stimulating factor 1 receptor (CSF1R) signaling regulates proliferation, differentiation, and function of macrophage lineage cells, such as microglia, and is involved in the regulation of homeostatic as well as inflammatory effects of microglia [19–22]. Pharmacologic inhibition of CSF1R has been reported to be beneficial in Alzheimer’s disease [21, 22]. The use of short hairpin RNA (shRNA) lentiviral particles allows the strategic and localized knockdown of target gene expression in vivo [23]. Lentiviral particles are widely used in central nervous system research since they are retroviruses that integrate in the host genome and can infect both dividing and non-dividing cells such as microglia [23]. Accordingly, the current study was designed as an initial step to begin exploring the possible connections between the elimination of microglia with shRNA-mediated CSF1R knockdown and the development of progressive PSCI in diabetic rats.

## Methods

### Animal model

Male Wistar rats (Envigo RMS, Inc., Indianapolis, IN) were housed in the animal care facility at Augusta University, which is approved by the American Association for Accreditation of Laboratory Animal Care. All experiments were conducted in accordance with the National Institute of Health (NIH) guidelines for the care and use of animals in research. Furthermore, all protocols were approved by the institutional animal care and use committee.

### Type 2 diabetes mellitus (T2DM) induction

Diabetes was induced by a high-fat diet/low-dose streptozotocin (HFD/STZ) combination as we previously reported [16]. Male rats were received at 4 weeks of age and immediately started and maintained on a 45% kcal fat diet for the remainder of the study (Research Diets Inc., New Brunswick, NJ). A single dose of STZ injection (35 mg/kg; Cayman Chemical, Ann Arbor, MI) was administered intraperitoneally (ip) at 6 weeks of age. If blood glucose was not above 150 mg/dL 5 days post-injection, a second small dose (20 mg/kg) was administered. Control rats were purchased at 10–11 weeks of age and maintained on regular chow with 4% kcal fat. Body weight and blood glucose were measured weekly.

### In vivo CSF1R silencing

In vivo CSF1R silencing was achieved by intracerebroventricular (ICV) injections using a stereotaxic instrument under isoflurane anesthesia. Stereotaxic coordinates used were − 1 mm anteroposterior, 2 mm lateral, and − 3 mm dorsoventral relative to bregma. A total of 2.5 μl of lentiviral CSF1R shRNA (SMARTchoice lentiviral rat CSF1R hCMV-TurboGFP shRNA, 1 × 108 TU/mL, Dharmacon, #NC1650992) or non-targeting control vector (NTC) was slowly injected bilaterally over 5 min into each of the lateral ventricles. In the first round of experiments, a shRNA package that included 3 slightly different constructs were injected into control animals to determine the efficiency of the constructs. Animals were then kept for 14 days to allow for recovery and viral particle integration into their genome. We have previously shown that at least 10 days are required to achieve 70% or higher knockdown [23]. The control animals were sacrificed after those 14 days to optimize the shRNA selection. After selecting the shRNA construct that achieved the greatest knockdown, the diabetic animals were injected with the NTC or CSF1R shRNA. Fourteen days later, the animals underwent MCAO surgery. The animals were tested for behavioral deficits during the recovery phase and 3 weeks post-MCAO; (5 weeks post-injection) the rats were sacrificed.

### Middle cerebral artery occlusion (MCAO) surgery

Rats were subjected to transient focal cerebral ischemia (60 min MCAO) or sham surgery at 12–15 weeks of age using 4–0 silicon-coated nylon suture (Doccol 403756). The rats were anesthetized using 2–5% isoflurane, a ventral mid-line neck incision was made, the right common carotid artery (CCA) was exposed and lightly tied, and the external carotid artery (ECA) was ligated and cut. The suture was marked at 1.8 and 2 mm then advanced from a nick at the ECA into the internal carotid artery (ICA) until positioned between the 1.8 and 2 mm marks, indicating the branching of the middle cerebral artery (MCA). The suture was tied in place for the duration of the occlusion and the rats were allowed to recover from anesthesia. At the end of the 60-min occlusion time, the rats were re-anesthetized, the suture was removed for reperfusion, and the small nick at the ECA was permanently ligated. In sham animals, there was no ICV injection and rats underwent sham MCAO in which the CCA was isolated and the ECA was cut and ligated without insertion of the suture. We began with a group of 25 animals but 3 animals died prior to stroke or ICV injection, resulting in a total of 22 animals that received either ICV injections followed by MCAO surgery or simply received sham surgery without ICV injection. A total of 8 NTC, 9 ShRNA, and 5 sham animals underwent MCAO or sham surgery. After including post-stroke mortality, the groups were as follows: NTC (*N* = 5), shRNA (*N* = 7), and sham (*N* = 5). One shRNA animal was excluded due to a failed injection resulting in *N* = 6 for the shRNA group. Two NTC animals were excluded from flow cytometry analysis due to use of the both hemispheres, while the other samples only evaluated the ipsilateral hemisphere.

### Assessment of sensorimotor function

Sensorimotor function was evaluated by the adhesive removal task (ART) as previously described [24]. This was performed at baseline, day 3 and weeks 1, 2, and 3. For the ART, the rats were trained for 4 days and then baseline measurements were recorded prior to stroke, but after ICV injection. Contact and removal latency of the adhesive paper dot was recorded and the average was taken from 3 trials with a maximum removal latency of 180 s per trial.

### Assessment of cognitive function

All behavioral assessments were conducted by a blinded investigator. Cognition was assessed by the 2-trial Y-maze and novel object recognition (NOR) to examine spatial and working memory, respectively. Rats were trained 4 days prior to baseline testing, which was conducted prior to stroke but after ICV injection. Tests were repeated at weeks 2 and 3 post-MCAO. In the first trial of 2-trial Y-maze, rats were allowed to freely explore 2 open arms for 10 min. The animal was returned to its home cage for a 45-min delay. In the second trial, rats were positioned in the start arm facing the end wall and allowed to explore all 3 arms of the Y-maze apparatus freely for 3 min. Total time spent in each arm was recorded. Multiple indices were calculated: percent time spent in the novel arm (time in novel arm divided by total time in all arms × 100) and percent forced alternation (FA) (percent of alternations starting in the novel arm as first arm in the alternation sequence) [25]. A successful alternation was defined as the animals going into different arms in three consecutive arm entries.

For NOR, animals were habituated for 10 min in the testing apparatus for 4 days prior to baseline testing. On testing days, the rats participated in 3 phases: acclimation, familiarization, and novel. In the acclimation phase, the animal was allowed to explore the apparatus for 5 min and then returned to his home cage. During the familiarization phase, rats were allowed to explore 2 identical objects placed equidistant from the walls with 20 cm between the objects for 5 min. After a 45 min delay in their home cage, the rat was allowed to explore a novel object paired with the familiar object for 5 min. Rats were started in the center of the testing apparatus for each session. Objects and the testing area were cleaned with vital oxide odor eliminator between each phase. The time spent exploring each object was recorded for the familiarization and novel phases. Multiple indices were calculated based on the total time (second) spent exploring novel object (*T*_N_) and familiar (*T*_F_) object: recognition index (RI = *T*_N_/(*T*_N_ + *T*_F_)), discrimination index 1 (d1 = *T*_F_ − *T*_N_), and discrimination index 2 (d2 = (*T*_N_ − *T*_F_)/(*T*_N_ + *T*_F_) [26].

### Euthanasia, specimen collection, and molecular techniques

Rats were euthanized 3 weeks post-MCAO or sham surgery using isoflurane overdose and cardiac puncture. They were then perfused with 50 mL PBS and the brains were extracted. Using a brain matrix, the brain was sliced from B, containing the prefrontal cortex, through slice D, containing the hippocampus, for a resulting 6 mm thick slice. The proximal portion of the B slice (1 mm) and the distal portion of the D slice (1 mm) were taken for immunocytochemistry (IHC) and then further sectioned to a thickness of 30 μm for IHC staining (Fig. 3a). The internal portion (4 mm) was taken for flow cytometric analysis.

### Flow cytometry

Following isolation of the ipsilateral hemispheres of B-D slice, containing the prefrontal cortex through the hippocampus, the tissue was minced into 1 mm^3^ pieces and was dissociated using Worthington’s Papain Dissociation kit (catalog number LK003153) with the following modifications: (1) tissue was left in dissociation medium for 15–25 min and (2) oxygen was continuously perfused over (not bubbled in) the solution for the duration of the incubation period [27]. Microglia were isolated as described below.

### Myelin debris removal and microglial isolation

A debris removal step was performed using modified protocols from Miltenyi Biotec’s Myelin Removal Kit (catalog number (Miltenyi Biotec, Germany) and CD11b^+^ Microbeads (Miltenyi Biotec, Germany)). Following dissociation, up to 10^7^ cells were suspended in 200 μl 0.5% BSA in PBS buffer and incubated with 20 μl anti-myelin microbeads for 15 min at 4 °C. The cells were then placed in the mini-MACS magnetic separator column and the clean supernatant was eluted out. The cells were then incubated with 20 μl of CD11b^+^ beads to isolate the microglia/macrophage population and isolated using the mini-MACS separator once again. CD11b^+^ cells were then further processed with surface and intracellular microglia makers.

### Cellular staining

Cells were incubated with surface markers against pre-conjugated antibodies CD45-APC (eBioscience, San Diego, CA) and CD86-FITC (BD bioscience, San Jose, CA) for 20 min. Cells were then permeabilized for intracellular staining with a fixation/permeabilization solution kit (eBioscience, San Diego, CA). Cells were then separated into two groups and incubated with markers CD206 (Abcam, Cambridge, MA), TNFα (BD bioscience, San Jose, CA), and IL-10 (BD bioscience, San Jose, CA), or TNFα (BD bioscience, San Jose, CA) and TMEM119 (Novus, Centennial, CO). Secondary antibodies PE (eBioscience, San Diego, CA) and PerCP (BD Bioscience, San Jose, CA) were used in both groups. Cells were then washed and analyzed using the Cytoflex (Beckman Coulter, Indianapolis, Indiana).

### Imaging and analysis

To minimize false-positive events in flow cytometry, the number of positive events detected with the negative staining control for each individual channel was subtracted from the number of positive cells stained with corresponding antibodies. In order to optimize the detection of the antibodies and minimize non-specific binding, AbC Total Antibody Compensation Beads were used in junction with negative single-color staining and unstained cell controls. Tight titration of each antibody was performed to ensure the highest positive signals in the target populations, while reducing spread in the negative population. Cells expressing a specific marker were reported as the number of gated events. The markers used are shown in Table 1. Microglia were first identified as CD11b^+^/CD45^+^ low. Pro-inflammatory microglia were further identified as CD86^+^/TNFα^+^; anti-inflammatory cells were identified as CD206^+^/IL-10^+^. Residential microglia versus infiltrating macrophages were also identified as CD11b^+^/CD45^+^ without separation of low versus high. Residential microglia were then further identified as TMEM119^+^, while infiltrating macrophages were identified as TMEM119^−^. Pro-inflammatory macrophages were then further identified as CD11b^+^/CD45^+^/TMEM119^−^/CD86^+^/TNFα^+^ cells.

**Table 1:** Flow cytometry markers utilized to identify particular cell populations

	CD11b	CD45	TMEM119	CD86	TNFα	CD206	IL10
M1 (CD86+/TNFa+)	+	+ low	N/A	+	+	N/A	N/A
M2 (CD206+/IL10+)	+	+ low	N/A	N/A	N/A	+	+
Residential microglia (TMEM119-)	+	+	+	N/A	N/A	N/A	N/A
Infiltrating macrophages (TMEM119-)	+	+	−	N/A	N/A	N/A	N/A
M1 macrophages (TMEM119-/CD86+/TNFa+)	+	+	−	+	+	N/A	N/A

### Immunohistochemistry (IHC)

Brains were extracted and post-fixed in 4% PFA overnight. Free-floating 30 μm sections were incubated overnight with anti-IBA-1 (ionized calcium-binding adaptor molecule 1, 1:500, Wako, Japan) and anti-GFAP (glial fibrillary acidic protein, Sigma-Aldrich, Burlington, MA) for B slice sections containing the PFC and with anti-MBP (Myelin Basic Protein, Abcam, Cambridge, MA) and NF200 (Neurofilament, Abcam, Cambridge, MA) for the D slice containing the cerebral cortex (CTX). Cells were then incubated with Texas Red and Alexa Flour 488-conjugated secondary antibodies (Cell Signaling Technology, Danvers, MA, USA) used at 1:200 for 2 h at room temperature. Nuclei were counterstained using DAPI (406-diamidino-2-phenylindole, Roche Basel, Switzerland) and sections were mounted on glass cover slips. Imaging was performed using the Keyence Microscope and Z stacked through the 30-μm thickness to obtain a complete count of the tissue area (Itasca, IL) for IBA-1 and GFAP quantifications. The counts of IBA-1 and GFAP were completed utilizing a double extraction method where only the cells that also co-stained for DAPI were counted. Sections were derived from a single plane for MBP and NF200 quantifications. A double extraction method was also utilized, where only the MBP that co-stained for NF200 was counted. The area of MBP:NF200 was then compared to derive a ratio of myelination.

### Statistical analyses

All behavioral tasks were analyzed by SAS© version 9.4 (SAS Institute, Inc, Cary, NC), and all molecular tests were analyzed by GraphPad Prism 8. One-way ANOVA was utilized to evaluate all of the molecular data. Tukey’s multiple comparison was evaluated from significant ANOVAs and is displayed on the graph. All graphs display error bars that represent the standard error of the mean (SEM). Behavioral tests were analyzed for group and time main effects as well as the interaction of group over time using repeated measure ANOVAs with measurements taken across time utilizing the last observation carried forward method for any missing data. A Tukey’s multiple comparison test was used to compare means from significant ANOVA results. Statistical significance was determined at a type I error rate of 5%.

## Results

### Bilateral injections of CSF1R ShRNA resulted in a significant reduction of both residential microglia and macrophages

**Fig. 1:**
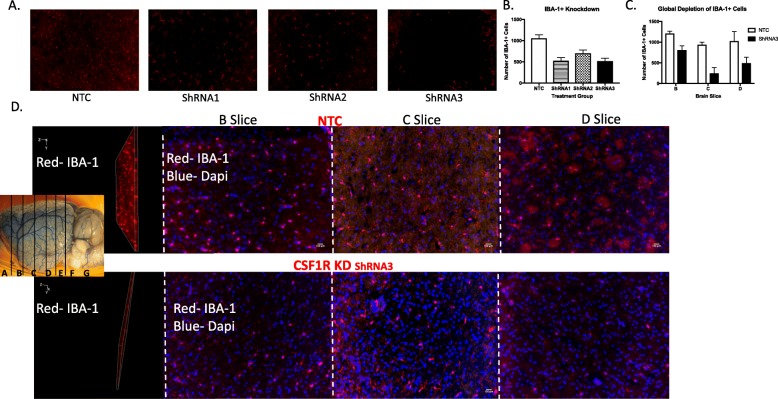
Bilateral CSF1R ShRNA injection resulted in a global knockdown of IBA-1+ cells brain 14 days post-injection. Images were derived from control animals and were Z stacked and quantified throughout the layer. Thirty-micrometer-thick sections were taken from the B, C, and D slice, both the layer and a representative image indicating the slides are illustrated in **a** and quantified in **b**. Three ShRNA constructs all targeted at the CSF1R were evaluated 14 days post-injection. Based on this evaluation, we choose the ShRNA with the best knockdown percentage to proceed with the study. Representative images of the chosen ShRNA and the quantification of the degree of knockdown are shown in C and D. ShRNA injection resulted in a reduction of IBA-1+ cells in the "slice" (**b**"**d**"), C slice (**"d"c**), and D slice (**"d"d**) (*N* = 4/group)

In order to identify the best shRNA construct to silence the CSF1R, 3 different shRNAs were injected into the lateral ventricles of normal Wistar rats. The knockdown percentages were then evaluated in the B, C, and D slice with IHC via IBA-1+ staining 14 days post-injection (Fig. 1a). Our shRNA of choice exhibited a global knockdown percentage of 72% as quantified by IBA-1+ cell counts 2 weeks after stroke. Specifically, there was a 48% knockdown in the B slice, 87% in the C slice, and 68% in the D slice (Fig. 1b).

**Fig. 2:**
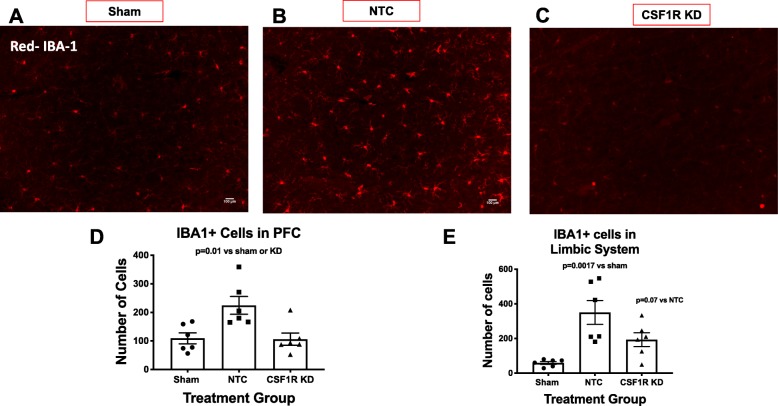
CSF1R KD resulted in a sustained knockdown in diabetic animals 3 weeks post-stroke and 5 weeks post-injection. **a**–**c** Depicts representative images of IBA-1+ cells in the PFC of slice B for NTC, CSF1R KD, and sham animals, respectively. **d** The PFC of diabetic animals have more IBA-1+ cells 3 weeks after a stroke. However, CSF1R KD lowered this upregulation. **e** Like the PFC, the limbic structures of diabetic animals also have more IBA-1+ cells 3 weeks after a stroke. One*-*CSF1R KD lowered this upregulation but still remained higher compared to the shams (*N* = 5–7/group)

After identifying a shRNA of choice, we evaluated whether the silencing would produce similar results in diabetic animals, and whether that knockdown would be sustained over 5 weeks. Fourteen days after shRNA injection, the animals underwent MCAO or sham surgery. Three weeks post-stroke (5 weeks post-injection), there was a 53% reduction in the PFC of the B slice and a 45% reduction in the limbic structure of the B slice (Fig. 2). The PFC of NTC animals exhibited an upregulation of IBA-1+ cells 3 weeks post-stroke (Fig. 2d). However, CSF1R silencing lowered this upregulation to sham levels (Fig. 2d). Like the PFC, the limbic structures of NTC animals also had more IBA-1+ cells 3 weeks post-stroke (Fig. 2e). In this case, CSF1R silencing prevented the upregulation, but it was still higher than that was observed in sham animals (Fig. 2e)*.*

**Fig. 3:**
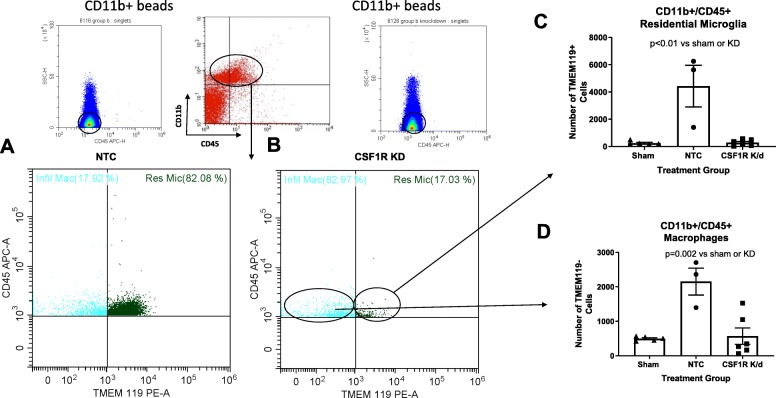
CSF1R KD resulted in a significant reduction of both residential microglia and macrophages. We gated for CD11b+/CD45+ cells indicative of both microglia and macrophages as depicted in **a** and **b**. **c** Three weeks post-stroke diabetic animals had an increase in residential microglia that CSF1R KD drastically lowered by 94%. **d** The macrophage infiltration after stroke was also lowered by 74% (*N* = 3–7/group)

We used flow cytometry to both quantify the knockdown globally and differentiate the residential microglia from the macrophages. CSF1R silencing resulted in a significant reduction of both residential microglia and macrophages. We gated for CD11b^+^/CD45^+^ cells, indicative of both microglia and macrophages as depicted in Fig. 3 a and b. We then separated these cells utilizing TMEM119, a residential microglia marker. Three weeks post-stroke, diabetic animals had an increase in residential microglia compared to sham animals and CSF1R KD resulted in a drastic 94% knockdown as measured by flow cytometry (Fig. 3c). The residual IBA-1+ cells measured in IHC of the CSF1R silenced animals actually derived from the infiltrating macrophages, as the injection only lowered this population by 74% post-stroke when evaluated by flow cytometry (Fig. 3d).

### CSF1R silencing improved survival after stroke

**Fig. 4:**
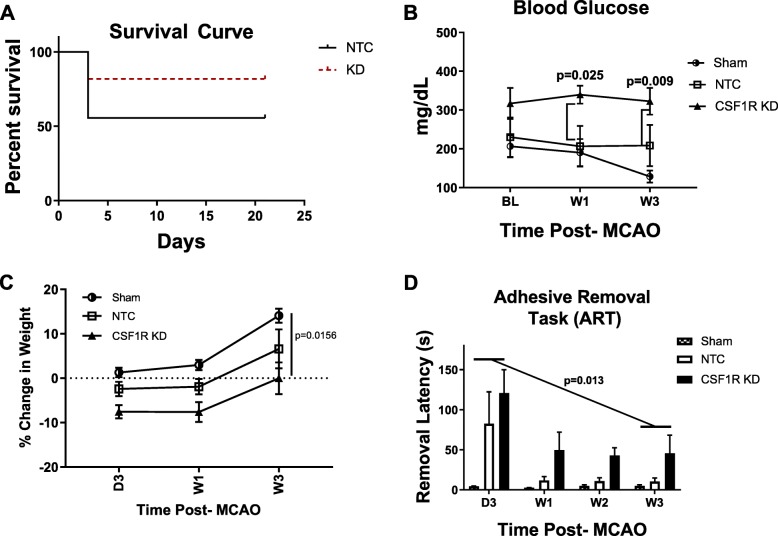
CSF1R KD improved survival. **a** CSF1R animals experienced a 22.2% mortality post-stroke, while the NTC animals experienced a 37.5% mortality. **b** Stroke in both NTC and CSF1R KD animals resulted in increased BG. **c** Body weight steadily increased over time in all groups. When all three groups compared, body weight was significantly different between the groups (*p* = 0.0156). However, there was no difference in percent body weight change in NTC and CSF1R KD groups that were subjected to stroke as compared to sham animals. **d** The adhesive removal task (ART) was utilized to measure sensorimotor function. Overall, there was an improvement in latency over 3 weeks (*p* = 0.013) and there was no significant difference between the NTC and CSF1R KD animals (*N* = 5–7/group)

CSF1R silencing reduced mortality (22.2%), compared to the NTC group (37.5%) (Fig. 4a). The high mortality in the NTC group is similar to the mortality seen in this diabetic stroke model without the ICV injections [28]. When blood glucose levels were compared at a given time point, there was no difference among the three groups (Fig. 4b). Analyses of the data across the weeks showed a trend for an interaction (*p* = 0.086) suggesting a difference among the groups most likely due to the observation that the sham group tailed off the last week. The percent change in body weight from baseline indicated a time effect with a decrease up to week 1 followed by an increase by week 3 in all groups (Fig. 4c). Since sham animals showed no deficiencies in fine sensorimotor skills as determined by ART, only NTC and KD groups were compared. There was a significant difference across the weeks with both groups showing improvement over time (*p* = 0.013). There did appear to be a trend (*p* = 0.081) toward worse performance on the ART in the CSF1R KD group, compared to the NTC group, but this did not achieve statistical significance. This may have been influenced by the excessive mortality in the NTC group, reducing the power of the observation.

### CSF1R silencing reduced inflammation

**Fig. 5:**
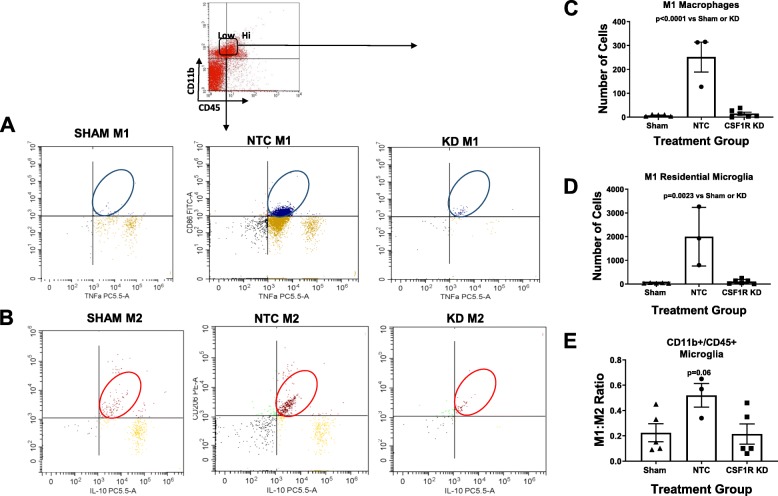
CSF1R KD lowered the amount of inflammatory microglia. We gated for M1 and M2 cells as indicated in **a** and **b**. **c** Three weeks post-stroke diabetic NTC but not CSF1R animals have an increased pro-inflammatory/anti-inflammatory ratio. **d** CSF1R KD lowered the number of pro-inflammatory microglia, and pro-inflammatory macrophages (**e**) (*N* = 3–7/group)

In an effort to further investigate the ramifications of CSF1R silencing, we then evaluated the impact it had on neuroinflammation. Since we previously observed that the modulation of the pro-inflammatory/anti-inflammatory ratio toward a more anti-inflammatory profile was able to preserve cognition, we evaluated the pro-inflammatory/anti-inflammatory ratio that persisted in the remaining microglia population that was not depleted. To evaluate this, we gated for CD11b^+^/CD45+ low cells. The expression levels of CD45 (low versus high) have previously been accepted as a way to distinguish between microglia and macrophages. Out of the CD11b^+^/CD45+ low population, we then gated for pro-inflammatory and anti-inflammatory cells (Fig. 5 a and b). We discovered that, 3 weeks post-stroke, diabetic animals that received the NTC displayed a chronically elevated pro-inflammatory/anti-inflammatory ratio compared to sham animals (Fig. 5c). The CSF1R KD animals however did not (Fig. 5c). CSF1R silencing lowered the number of pro-inflammatory residential microglia that remained chronically elevated in the NTC group (Fig. 5d). CSF1R silencing also lowered the number of pro-inflammatory infiltrating macrophages which too were chronically elevated after a stroke (Fig. 5e).

**Fig. 6:**
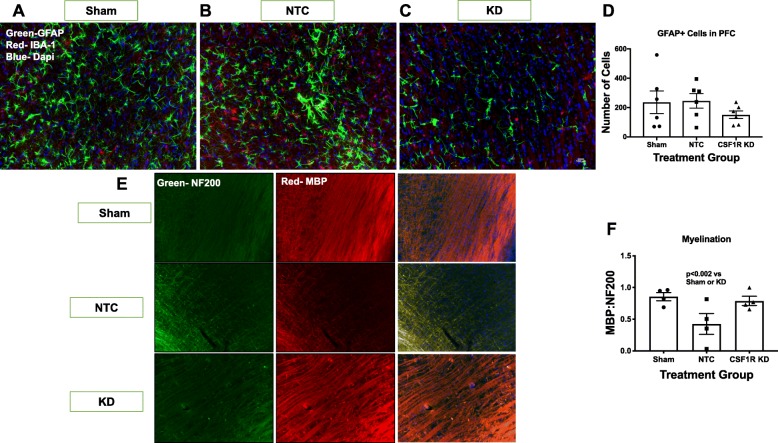
CSF1R KD improved myelination. **a**–**c** Depicts representative images of GFAP+ cells of slice B for sham, NTC, CSF1R KD animals, respectively. **d** There was no significant difference in the number of GFAP+ cells in the PFC or limbic structures of slice B and GFAP+ cells. **e** Depicts representative images of NF200+ and MBP+ cells of slice B for NTC, CSF1R KD, and sham animals, respectively. Using MBP to stain myelin and NF200 to stain the axons, we evaluated the ratio of MBP:NF200 as a measure of myelination (**e**). Although diabetic animals exhibited demyelination 3 weeks after stroke, CSF1R KD prevented this decline in myelination (*N* = 5–7/group)

GFAP^+^ cells are astrocytes that also play an essential role in the neuro-inflammatory response to stroke. With this in mind, we evaluated the impact of CSF1R silencing on astrocyte cell number in both the PFC as well as the limbic structures of the B slice (Fig. 6a–c). The limbic structures contained more GFAP^+^ cells than the PFC. There was no significant difference in the number of GFAP^+^ cells between groups in either region (Fig. 6d).

Demyelination is a serious consequence of chronic inflammation. Although CSF1R silencing lowered the presence of inflammatory cells to alleviate inflammation, the anti-inflammatory microglia have been shown to play a significant role in remyelination post-stroke [11]. Thus, we wanted to evaluate the impact of CSF1R silencing on myelination of the cerebral cortex (CTX), a region of white matter in the rat [11]. Using MBP to stain myelin and NF200 to stain the axons, we evaluated the ratio of MBP:NF200 as a measure of myelination (Fig. 6e). The NTC animals exhibited extensive demyelination 3 weeks post-stroke (Fig. 6f). CSF1R silencing preserved the myelination in the CTX post-stroke, as the neurons were significantly more myelinated compared to the NTC (Fig. 6f). CSF1R silencing was able to lower the presence of inflammatory cells and halt a consequence of chronic inflammation after stroke.

### CSF1R silencing may preserve cognitive function

**Fig. 7:**
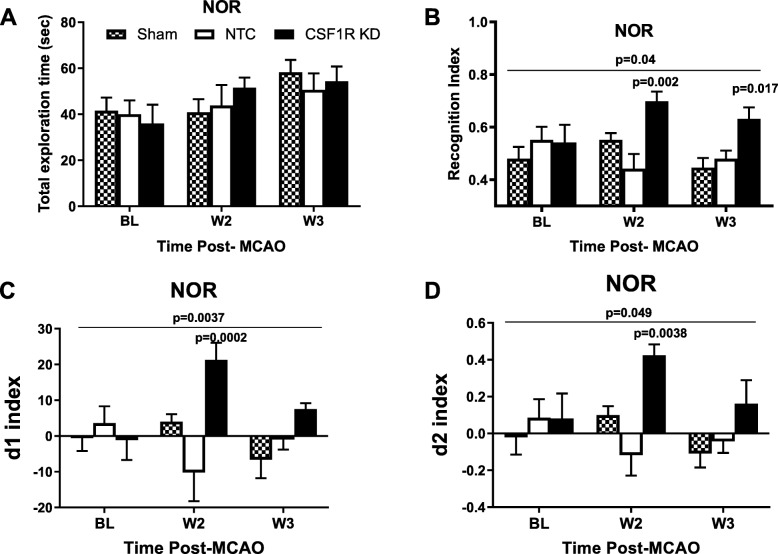
CSF1R KD preserved cognition post-stroke. **a** There was no difference in object exploration time between groups. **b**–**d** CSF1R KD preserved working memory as measured by NOR recognition index (**b**), discrimination index d1 (**c**), and discrimination index d2 (**d**). Comparison of means over time indicated an interaction for all three indices showing an increase (improvement) only in the KD group. Significant differences observed at weeks 2 and 3 are also shown on the graphs (*N* = 5–7/group)

**Fig. 8:**
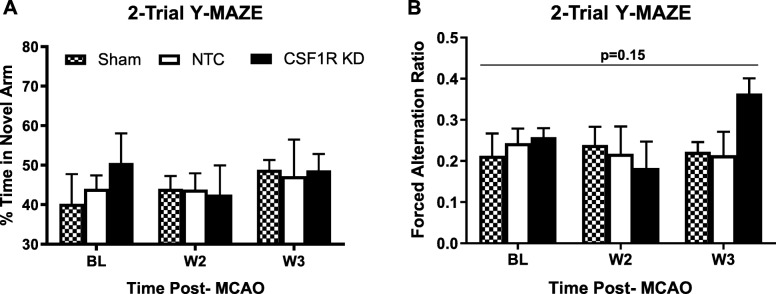
Stroke does not impair the ability to recognize the novel arm in 2-trial Y-maze. **a** There was no difference in percent time spent in the novel arm between groups. However, forced alternation (FA) ratio (**b**) appeared to improve only in the KD group. A successful alternation was defined as the animals going into different arms in three consecutive arm entries and FA ratio was expressed as the number alternations starting in the novel arm divided by total successful alternations (*N* = 5–7/group)

Our lab has previously shown that cognitive deficits were improved in diabetic animals when microglia polarization was modulated pharmacologically and chronic inflammation was relieved [16]. Thus, we evaluated the impact of global microglia knockdown on cognition after stroke. The total exploration time in NOR was similar between the groups (Fig. 7a). CSF1R silencing prevented the decline in working memory as indicated by multiple indices in NOR. Analysis of the means for RI showed an interaction such that KD increased RI at weeks 2 and 3 while there were no differences across weeks for sham or NTC groups (Fig. 7b). When compared at each time point, there was no difference at baseline but KD group had significantly higher RI at week 2 and week 3 than the other groups. d1 and d2 indices indicated similar improvement with CSFR1 silencing as indicated by an interaction over time resulting in higher d1 and d2 indices in the knockdown group as compared to sham and NTC groups (Fig. 7 c and d). 2-trial Y-MAZE was employed to evaluate spatial memory. There was no difference in the percent time spent in the novel arm (Fig. 8a). While it did not reach significance, the forced arm alternation ratio in which the animals started a correct alternation of arms in the novel arm improved by week 3 only in the knockdown group (*p* = 0.15, Fig. 8b).

## Discussion

Sustained neuroinflammation is a critical pathogenic feature of cognitive impairment due to Alzheimer’s disease and related dementias [29]. We have reported that sustained microglial activation that occurs in both hypertensive [30] and diabetic stroke models is associated with progressive post-stroke cognitive impairment [13, 16]. These findings led us to postulate that microglia are responsible for the progression of cognitive impairment after stroke in diabetes. In order to test this, we used intracerebroventricularly injected shRNA to silence CSFR1 and deplete microglia in diabetic animals prior to MCAO and followed them for 3 weeks. Here, we provide encouraging exploratory evidence that CSF1R silencing dampened inflammation and prevented the progressive cognitive deficits due to stroke.

Although microglial depletion has been reported to reduce neuroinflammation leading to improved disease phenotype in several mouse models of neurodegenerative diseases, it has previously been shown to have detrimental effects on stroke outcome in healthy animals [19, 21, 31]. A study utilizing oral administration of PLX3397 to silence the CSF1R in rats prior to stroke reported that it exacerbated stroke severity and actually increased the infarct size at 3 days by increasing the production of inflammatory mediators by astrocytes (IL-1b, iNOS, IL-6). Since the anti-inflammatory microglia function to restrict the ischemia-induced astrocyte response and provide neuroprotective effects, this feature was lost upon their depletion [32]. Using similar methods, another study reported a drastic 60% increase in infarct size with microglial depletion. In that study, focusing on the first 24 h after ischemia, CSF1R silencing resulted in increased excitotoxicity, dysregulated calcium (Ca^2+^) responses, and a complete loss of spreading depolarization [31]. Since astrocytes play an essential role in Ca^2+^ regulation in neurons and protection from excitotoxicity, it is likely that the altered astrocytic response in the absence of microglia contributed to the acute injury. These findings are consistent with earlier evidence, garnered from a genetic hippocampal lesion model, where depletion of microglia during the lesioning worsened the injury, but depletion in the chronic phase lessened inflammation and subsequent neuronal loss [33]. Our study is the first to evaluate the impact of CSF1R silencing on longer-term stroke behavioral outcomes under a comorbid neuroinflammatory condition, diabetes. Unlike the previous studies where the acute lesion size was increased, we demonstrated preserved working memory at 3 weeks, and this was accompanied by increased myelination in the white matter of the animals. This could be due to the fact that diabetic animals have a dysfunctional anti-inflammatory microglia response, as discussed below, thought to be responsible for the early protective effects, so depletion does not impact the beneficial effects of microglia post-stroke [11].

Anti-inflammatory microglia have been reported to peak 3 days after a stroke and play an essential role in synapse remodeling through the release of microglia-derived BDNF [6, 17]. They additionally play an essential role in synapse transmission through the remyelination of axons after a stroke via the modulation of oligodendrogenesis [11, 34]. However, chronically active microglia have been shown to be responsible for the late demyelination occurring after stroke [35]. Diabetic animals have been found to have particularly impaired myelination post-stroke, consistent with their increased pro-inflammatory activation, and lack of acute anti-inflammatory response [11]. Although microglia support healthy synaptic pruning, brain connectivity, and development, homeostatic functioning is lost in neurodegenerative diseases [21]. Unfortunately, the chronic inflammation in diabetes is similar to that observed in neurodegenerative diseases, outweighing any potential benefits of alternatively activated (M2) microglia.

With the bilateral ICV administration, we noted a 94% reduction in TMEM119^+^ residential microglia 5 weeks post-injection and 3 weeks post-stroke as measured by flow cytometry. This was similar to the 87% reduction that we observed using IHC to quantify the number of IBA-1+ cells throughout the B–D slice in control animals 14 days post-injection. With this, we are confident that administering the CSF1R-targeted ShRNA 14 days prior to MCAO surgery allowed enough time for integration of the ShRNA and for the full knockdown to be complete prior to ischemic insult. It is interesting that although there was a drastic reduction of residential microglia throughout the B-D slice, there was only a 45–53% reduction in the B slice as measured with IHC in diabetic animals. This was similar to the 48% reduction observed in the control animals during the optimization phase. This may be due to the placement of the B slice in location to the lateral ventricles and an artifact of the limited diffusion potential of the viral vector. It is interesting that although the PFC within the B slice received the lowest percentage of knockdown relative to the C and D slice, the NOR task, which evaluates working memory and is predominantly dependent upon the PFC, was significantly improved. This suggests that the partial but not complete knockdown of microglia within that region was enough to improve cognitive deficit post-stroke. We did not observe any decline in spatial memory after stroke as determined by 2-trial Y-maze. This was different that our previous study in which we detected a progressive decline in percent time spent in the novel arm in diabetic animals when monitored up to 8 weeks post-stroke [16]. This may have resulted from differences in stroke severity. While we used a 60-min MCAO in both studies, in the previous study we employed a pharmacological intervention after stroke and used strict stroke inclusion criteria based on percent weight loss and changes in ART latency at day 3 post-stroke to randomize the animals to vehicle or treatment groups. This tactic resulted in a more consistently impaired cohort of animals. In the current study, since we injected viral vectors prior to stroke surgery, there was no second randomization after MCAO. Despite a lack of decline in spatial memory after stroke, the KD group shows better spatial recognition by week 3, strongly suggesting that microglia depletion improves this aspect of cognition as well.

Our lab has previously shown that diabetic animals have a 50% increase in IBA-1+ cells compared to control animals 8 weeks post-stroke, with the majority of this increase occurring in the infiltrating macrophage population [16]. Although there was a 94% knockdown of residential microglia, there was only a 74% knockdown of infiltrating macrophages in this study. We learned from previous studies that 8 weeks post-stroke, diabetic animals had far more IBA+ cells than control animals, but the increase derived from an increase in macrophages. Seventy-one percent of CD11b^+^/CD45^+^ cells in the PFC of diabetic animals were derived from infiltrating macrophages, while only 26% were derived from infiltrating macrophages in control animals [16]. The control animals in that study which exhibited a lesser degree of macrophage infiltration also displayed less functional and cognitive deficits. With the massive accumulation of macrophage infiltration in diabetic animals, the 74% knockdown may have restored balance to the diabetic brain, lowering the level of macrophage infiltration to that similar to what has been observed in our control animals.

The high degree of macrophage infiltration in the diabetic animals brings into question the blood brain (BBB) integrity. Utilizing the same HFD/STZ model reported here, our lab has previously shown that diabetic animals have a compromised BBB that persists 14 days post-stroke [13]. This brought into question whether the CSF1R silencing would improve or exacerbate the compromised BBB. Although our study was not able to address that directly, we can infer from the dampened percentage of macrophage infiltration (74% reduction) and the reduced inflammation that it may in fact improve, not exacerbate BBB damage. A study utilizing an orally administered CSF1R inhibitor similarly reported that there were no BBB changes that accompanied the depletion of microglia [31]. On the contrary, other studies targeting the CSF1R have reported that microglia depletion was associated with exacerbated BBB damage and peripheral cell infiltration [18, 36]. Some differences may arise from the timing of the depletion with various developmental stages. While damage to the BBB was detected when administered in young mice 2 weeks post-natal, it was not reported in 12–16-week-old mice [17, 32]. Another variable may be that the 12–16-week-old mice were analyzed post-stroke. An ischemic event can independently induce BBB alterations and may mask any potential BBB damage induced by the microglia depletion. In our study, diabetes and stroke both independently alter the BBB [12].

We utilized a recently identified marker, TMEM119, to differentiate infiltrating macrophages from residential microglia. This marker has recently been identified as a homeostatic marker for residential microglia. There is an emerging evidence that suggests that damage associated microglia (DAMs) have been shown to decrease in expression of TMEM119 in association with the development of Alzheimer’s disease [37]. With this in mind, under neurodegenerative conditions, such as diabetic PSCI, the use of TMEM119 to differentiate residential versus infiltrating macrophages may be skewed by the number of DAMs that lose their TMEM119 expression. It may also be true that what they evaluated in that study was actually an increase in infiltrating macrophages with disease progression. Further studies are warranted in this regard to validate the efficacy of TMEM119 in neurodegenerative conditions.

Although we expect the CSF1R silencing to be localized to the brain, a limitation of our study is that we did not evaluate the possible peripheral effects of this injection. A study that used pharmacological intervention with 3 weeks of oral administration of PLX3397 reported that the particular CSF1R inhibitor was specific to microglia and did not impact the macrophage population in the splenic tissue [31]. On the contrary, a different study that also used PLX3397 administration for 3 weeks reported that diabetic mice exhibited a decrease in peripheral macrophages [38]. This study demonstrated that CSF1R silencing reduced inflammation in adipose tissue. Interestingly, it downregulated the pro-inflammatory/anti-inflammatory ratio both in the blood and in visceral adipose tissue without impacting BG in those animals [31]. It completely depleted the number of circulating pro-inflammatory macrophages, without altering the anti-inflammatory. Since these approaches were administered orally while this current study was administered with ICV injection, one would expect a larger potential to impact the peripheral system compared to the localized injection in the brain. An interesting observation is that in our study, the KD and NTC groups had consistently higher blood glucose when compared to sham diabetic animals. This may be a result of the ischemic stroke induced in these animals. The fact that cognition was preserved only in the KD group despite elevated blood glucose suggests that reduction of inflammation by microglia depletion is an effective strategy to prevent progressive cognitive decline in comorbid disease models.

Alteration of the pro-inflammatory/anti-inflammatory ratio in diabetic animals may be beneficial both in the central and the peripheral nervous systems. Our lab previously showed that the lowering of this pro-inflammatory/anti-inflammatory ratio with oral administration of an angiotensin II type 2 receptor agonist, C21, was beneficial to functional recovery and the prevention of PSCI [16]. Although anti-inflammatory microglia are known for their role in tissue repair, the function of anti-inflammatory microglia in diabetic subjects should be evaluated for the retention of this characteristic. A recent study reported increased heterogeneity in the response subtypes of microglia in the context of neurodegeneration [39]. This was also observed in our diabetic animals that developed PSCI where we reported a massive overlap of CD206^+^ anti-inflammatory cells that also expressed a pro-inflammatoryCD86^+^ marker [16]. This increased heterogeneity in microglia markers and possibly, function, allows justification for a silencing approach since the microglia response with the diabetic subjects may be aberrant.

This study is not without limitations. This study serves as an initial step to exploring the role that microglia elimination may play in stroke recovery under comorbid conditions. Although this data is encouraging, it is exploratory in nature and not explanatory, as the small sample size is a limiting factor. Future studies are warranted to investigate the role of various cytokines and trophins such as BDNF and the various dynamic states of microglia that may be present. This study did not investigate the impact that the elimination of microglia may have on diabetic female rats, but future studies on such are warranted. Diabetic rats often suffer an extensive mortality after stroke, and this is amplified in female rats. Now that the basis of investigation has been set, future studies in female rats should be investigated. Lastly, due to the small sample size and long-term nature of the experiments, we were not able to evaluate infarct size. Additional studies of this effect are also warranted to illustrate a full picture of how exactly microglia elimination impacts diabetic stroke outcomes.

## Conclusions

Although CSF1R silencing has been reported to exacerbate stroke injury in previous experimental stroke studies, it actually lowered inflammation and improved survival and cognition when diabetes was present. These data strongly support a causal role of activated microglia in the development of PSCI in diabetic animals.

## Data Availability

All data will be made available from the corresponding author, with a reasonable request.

## Abbreviations

AT2R: Angiotensin II type 2 receptor
ART: Adhesive removal test
BDNF: Brain-derived neurotrophic factor
CCA: Common carotid artery
CSF1R: Colony stimulating factor 1 receptor
CTX: Cortex
ECA: External carotid artery
FA: Forced alternation
GFAP: Glial fibrillary acidic protein
HFD: High-fat diet
IBA-1: Ionized calcium-binding adaptor molecule 1
ICA: Internal carotid artery
ICV: Intracerebroventricular
IHC: Immunohistochemistry
KD: Knockdown
M: Microglia
MCAO: Middle cerebral artery occlusion
MBP: Myelin basic protein
NF: Neurofilament
NOR: Novel object recognition
NTC: Non-target control
PFC: Prefrontal cortex
PSCI: Post-stroke cognitive impairment
RI: Recognition index
shRNA: Short harpin RNA
VCID: Vascular contributions to cognitive impairment and dementia
